# Disrupted pursuit compensation during self-motion perception in early Alzheimer’s disease

**DOI:** 10.1038/s41598-017-04377-2

**Published:** 2017-06-22

**Authors:** Jingru Wang, Xiaojun Guo, Xianbo Zhuang, Tuanzhi Chen, Wei Yan

**Affiliations:** Department of Neurology, Liaocheng People’s Hospital and Liaocheng Clinical School of Taishan Medical University, Liaocheng city, Shandong Province 252000 China

## Abstract

Our perception of the world is remarkably stable despite of distorted retinal input due to frequent eye movements. It is considered that the brain uses corollary discharge, efference copies of signals sent from motor to visual regions, to compensate for distortions and stabilize visual perception. In this study, we tested whether patients with Alzheimer’s disease (AD) have impaired corollary discharge functions as evidenced by reduced compensation during the perception of optic flow that mimics self-motion in the environment. We asked a group of early-stage AD patients and age-matched healthy controls to indicate the perceived direction of self-motion based on optic flow while tracking a moving target with smooth pursuit eye movement, or keeping eye fixation at a stationary target. We first replicated the previous findings that healthy participants were able to compensate for distorted optic flow in the presence of eye movements, as indicated by similar performance of self-motion perception between pursuit and fixation conditions. In stark contrast, AD patients showed impaired self-motion perception when the optic flow was distorted by eye movements. Our results suggest that early-stage AD pathology is associated with disrupted eye movement compensation during self-motion perception.

## Introduction

In addition to the progressive loss of memory and cognitive functions, patients with Alzheimer’s disease (AD) at the early stage often exhibit declined visuospatial capabilities^1, 2^. At the behavioral level, it has been shown that AD patients have poor performance in spatial navigation tasks^3^ and impaired perception of optic flow that mimics self-motion in the environment^4, 5^. Comparing to healthy elderly individuals, AD patients possess elevated optic flow thresholds^6^ and are worse at perceiving and steering in the direction of self-motion based on the pattern of optic flows^7^. Consistent with these behavioral deficiencies, imaging studies have revealed that AD patients have noticeable atrophy in the superior parietal lobules^8, 9^ and they show reduced activations in dorsal visual stream areas that are implicated in visuosptial processing^10–12^. There is also evidence that AD patients have impaired ability to bind distinct visual features of a stimulus (e.g., color and motion) in a sensory integration task, implicating that AD pathology might be associated with declined cross-cortical connectivity in the cerebral cortex^13, 14^.

Corollary discharge, one special form of functional connectivity among cortical regions, is often referred to as efference copies of motor command signals sent from motor to sensory areas that are used to predict the sensory consequence of an impending movement^15, 16^. Corollary discharge is an integral part of cortical functions that allow the brain to monitor its own actions^17^ (e.g., during motor planning^18^ and motor executing^19^) and compensate for visual distortions to achieve perceptual stability in the presence of eye movements^20, 21^. There is growing evidence that corollary discharge functions might have failed in several clinical populations leading to neurologic symptoms^22^ and delusional experiences such as auditory hallucination^23^, mirror-touch synaesthete^24^, and schizophrenic disorders^25^. Up to date, there is little evidence of corollary discharge dysfunctions in AD pathology in the literature.

The purpose of this study is to address whether deficits in corollary discharge functions are present in patients at the early stage of AD. We will assess the influence of eye movements on optic flow perception in AD patients. Eye rotations during pursuit eye movement distort the optic flow by adding a rotational component to it^26^. Nevertheless, previous findings have shown that healthy human observers barely experience a twisted visual representation^27, 28^, owing to pursuit compensation mechanisms which are widely believed to rely on corollary discharge signals sent from pursuit areas to visual areas to cancel the distortion^29, 30^ (for an alternative view, please see^31^). In this study we will exploit the pursuit compensation effects to examine the potential dysfunction of corollary discharge in AD patients. To this end, we will compare the performance of optic flow perception between a group of AD patients and age-matched healthy elder participants, under the condition of either smooth pursuit eye movements or pure fixations.

## Material and Methods

### Subjects

Two groups of participants took part in the current study. The first group consisted of 15 early-stage AD patients who were selected based on two criteria: (1) They had to met the National Institute of Neurological and Communicative Disorders and Stroke-Alzheimer’s Disease and Related Disorders Association criteria for the diagnosis of AD^32^; and (2) According to the assessment of the Mini-Mental State Exam (MMSE)^33^, they had a MMSE score less than 25 but larger than 18, hence classified as mild cognitive impairment patients. The second group involved 15 age- and sex- matched healthy elderly, most of whom were the spouse or relatives of AD patients tested in the current study. All participants had passed the ophthalmologic exams to be free of other neurologic, ophthalmologic, or psychiatric illnesses. All participants had given informed consent for their participation in the current study prior to the experimentation. All the experiments were performed in accordance with the guidelines and regulations approved by the Ethics Committee of Liaocheng People’s Hospital, China.

### Apparatus and optical flow

Subjects seated in a dimly lit room. They rested their chins on a chinrest which helps to stabilize head position. They faced a CRT monitor (screen resolution: 800 × 600; refresh rate: 100 Hz) which was 57 cm away from the chinrest. We used the Psychophysical Toolbox^34, 35^ to generate the visual optic flow and design the control of experimental flow as described below. The optic flow was made of a cloud of moving dots, consisted of 2000 white dots (each 0.2 deg in diameter) on a black background (0.7 cd/m^2^) and distributed in a virtual trapezoidal volume. Dots moving outside the trapezoidal volume were randomly assigned to locations on the furthest base as the new starting locations. The speed and luminance gradients of moving dots within this column resemble the visual experience of forward translation in the environment at a speed of 2.5 m/s. Specifically, the focus of expansion (FOE) in the optic flow corresponds to the direction of self-motion, which was interleaved from trial to trial along an imaginary horizontal line, locating at one of the six possible positions (±6.4 degrees, ±3.2 degrees, and ±1.6 degrees), relative to the straight ahead direction (“dead ahead’).

**Figure 1:**
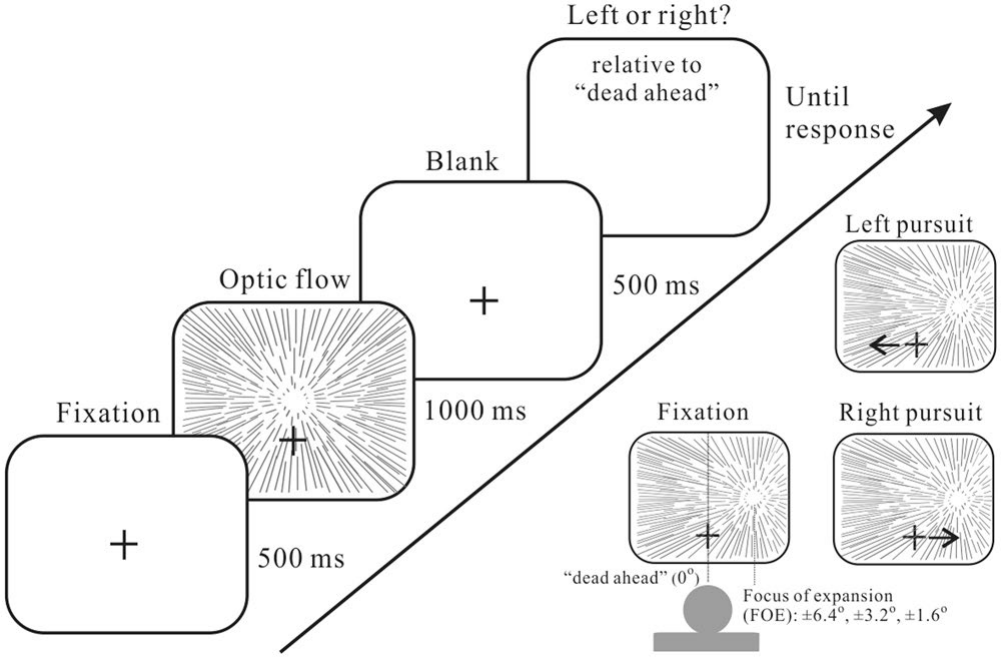
Optic flow perception task and experimental conditions. Participants faced a CRT monitor displaying a cloud of moving dots mimicking the observer’s forward translation on a straight path. The focus of expansion (FOE), which determines the direction of self-motion, could be at one of the six potential positions in a single trial. Participants were asked if perceived direction is to the left- or right- side of the dead ahead. In the fixation condition participants kept their gaze constant whereas in the pursuit conditions they made pursuit eye movements to track the moving cross.

Each trial begun with an initial ocular fixation at the cross (+) for 500 ms. Then the optic flow was presented for 1000 ms, and after a fixed delay of another 500 ms following optic flow offset, participants were required to report the perceived self-motion direction (either left or right relative to the straight ahead direction) by means of keyboard presses (Fig. 1). There were two task conditions. In the fixation condition, the central cross remained stationary during the visual stimulus presentation, and subjects’ task was to fixate their gaze at the cross. In the pursuit condition, during stimulus presentation the cross moved along the horizontal line at a speed of 5 degree/s (either leftward or rightward), and participants were asked to make smooth pursuit eye movements to keep track of the cross. Fixation and pursuit trials were randomly interleaved on a trial-by-trial basis. We used the method of constant stimuli and repeated each combination of conditions 10 trials to obtain the psychometric curve of self-motion perception as a function of FOE angles.

The logic in our design was, if the brain is able to compensate for visual distortions caused by pursuit eye movements, we should expect similar performance of self-motion perception between fixation and pursuit conditions. Many previous studies have already demonstrated that healthy observers can precisely judge self-motion directions in the presence of eye movements^27, 28^. So, the critical aspect in the current study was to see how the AD patients would behave in these two task conditions. Comparing the performance of optic flow perception between AD patients and healthy controls will then allow us to infer the functionality of corollary discharge signals in AD pathology.

### Data analysis

All data analysis was performed with custom-made MatLab scripts (The MathWorks, Natick, MA). To quantitatively characterize the performance of optic flow perception, we fitted a logistic function to the psychometric curve obtained for each subject. The logistic function was formulated as the following:

$$f(x)=\frac{1}{1+\exp (-\frac{1}{\sigma }(x-\mu ))}$$

where *x* was the FOE angle and *f*(*x*) was the proportion of rightward choice. Of the parameters derived from the fitted function, *μ* represented the FOE angle at which the subjects show equal left/right choice probability and was defined as the point of subjective equality (PSE), also known as the perceptual bias. The scaling factor *σ* (sigma) was inversely related to the slope of the psychometric function and was an index of perceptual sensitivity.

## Results

**Figure 2:**
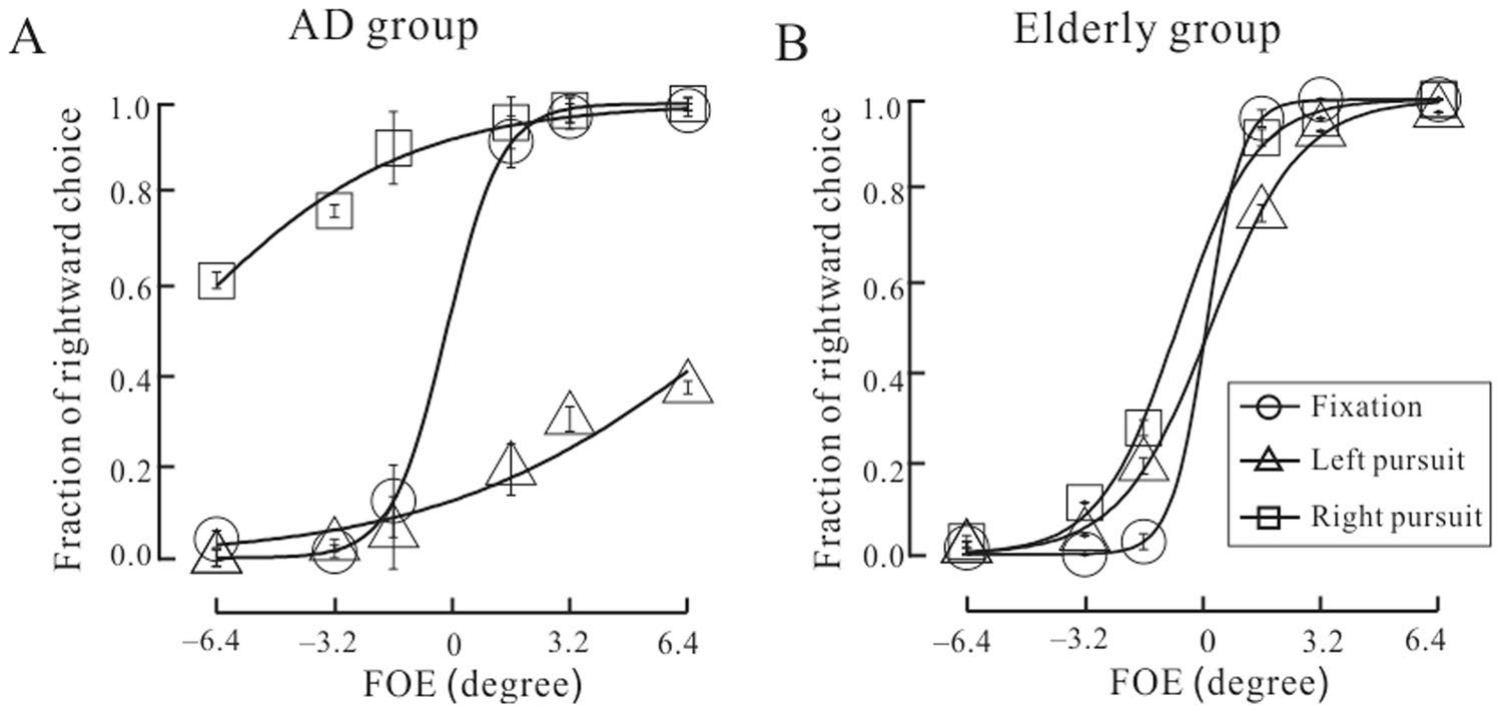
Psychometric performance of self-motion perception in the fixation and pursuit conditions for AD patients (**A**) and healthy elderly participants (**B**). Error bars denote mean and SEM.

We assessed the behavioral performance of optic flow perception in both pursuit and fixation conditions in AD patients and healthy elderly respectively, as illustrated in Fig. 2A,B. It was clear that the perception of optic flow differed dramatically between AD and elderly groups in the pursuit conditions. Specifically, we found that healthy individuals can accurately judge the direction of self-motion in the presence of pursuit eye movements. This was evident from the similar performances between pursuit and fixation conditions in the elderly group (Fig. 2B), which was consistent with previous studies^27, 28^. In sharp contrast, eye movements affected the perception of self-motion in AD participants (Fig. 2A). They performed poorly in the pursuit conditions comparing to the fixation condition. They were more likely to perceive a leftward self-motion in trials when the eyes move to the left (triangles), and vice versa for rightward pursuit movements (squares). In other words, self-motion perception in the presence of pursuit movements was biased in the same direction as the shift of FOE in the distorted optic flow. This means, AD patients exhibited disrupted eye movement compensation during optic flow perception.

**Figure 3:**
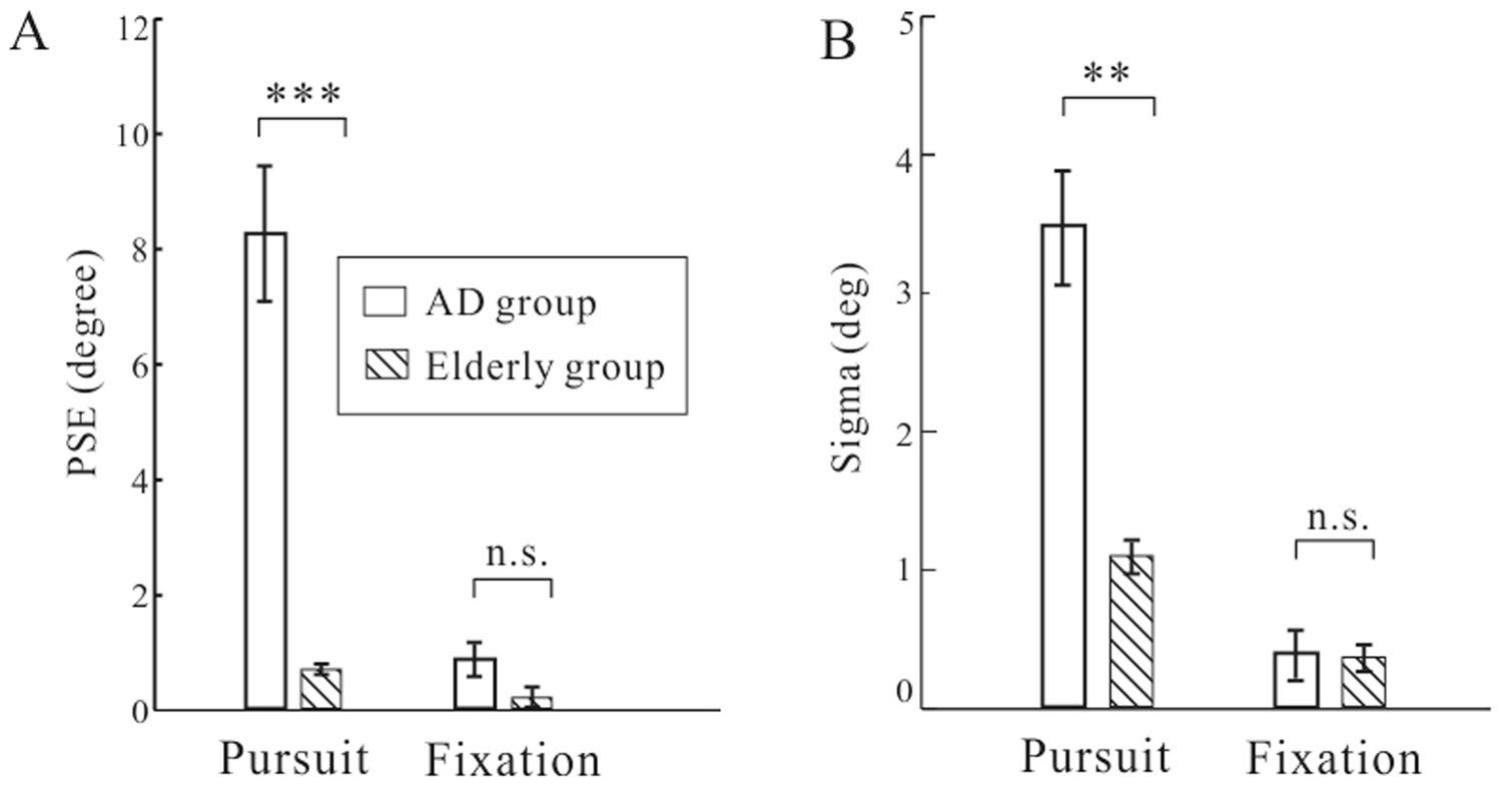
Averaged perceptual bias (indicated by PSE) and perceptual sensitivity (indicated by Sigma) derived from the psychometric function for AD patients (**A**) and healthy participants (**B**) in each condition. Error bars denote mean and SEM.

To quantitatively characterize these effects, we fitted a logistic function to the psychometric data and we did this for each task condition and for each observer, before we average them across participants (see Methods). Of the derived parameters, the absolute value of point of subjective equality (PSE) denotes the magnitude of perceptual bias whereas sigma measures the perceptual sensitivity. Figure 3A,B summarized the cross-participant mean and SEM of PSE and sigma values for each task condition in each group. Firstly, the perceptual biases in the pursuit conditions were significantly larger in AD patients (Mean ± SEM: 8.26 ± 1.14 degree) than those in the control group (0.69 ± 0.08 degree) (Fig. 3A). Unpaired *t*-test revealed that there was a significant difference between the two groups (p < 0.001). In contrast, the perceptual biases in the fixation condition were both very low (AD: 0.83 ± 0.35 degree; Elderly: 0.24 ± 0.17 degree) and they were not statistically different between groups (unpaired *t*-test, p > 0.05). Meanwhile, analysis of sigma also showed a similar pattern of results (Fig. 3B). AD patients had significantly larger sigma values than healthy controls in the presence of pursuit eye movements (p = 0.003, unpaired *t*-test). When there was no pursuit movement (fixation condition), mean sigma became statistically indistinguishable between groups (p > 0.05, unpaired *t*-test).

To sum it up, our results revealed that AD patients possessed larger biases and weaker sensitivity during the perception of distorted optic flow in the presence of eye movements, comparing to healthy elderly participants. When there was no distortion in the optic flow (fixation condition), self-motion judgments were not different between groups.

## Discussion

The current study revealed that AD pathology is associated with disrupted eye movement compensation during the perception of optic flow. We showed that both the sensitivity and bias of optic flow perception were impaired in AD patients in the presence of pursuit eye movement. However, AD patients did not exhibit deficits in the perception of optic flow when there was no eye movement. These findings add to the notion that non-cognitive impairments, such as sensory and motor dysfunctions, are increasingly evident in early-stage AD patients.

Optic flow perception is one commonly used perceptual task in AD research^2, 9^. Most previous studies focused primarily on the perception of optic flow during ocular fixation (without eye movements), that is, no corollary discharge is involved. For example, studies from Duffy laboratory report that AD patients show elevated optic flow coherence thresholds during passive viewing^5, 36^. Here we examined optic flow perception in AD patients in the presence of pursuit eye movements as well as during passive viewing (fixation condition). By comparing results across participant groups and task conditions, we can infer if corollary discharge of pursuit compensation during optic flow perception is intact in AD patients. To our knowledge, this is the first study that links corollary discharge dysfunction to AD pathology. We showed that AD patients exhibited impaired pursuit compensation during optic flow perception as comparing to healthy controls. In contrast, the performance of optic flow perception (both bias and selectivity) in the passive viewing condition (fixation trials) did not differ between groups. This ruled out a general decline of low level processing as the explanation for the observed results. Instead, the between-group difference in the optic flow perception was specific to eye movement compensations (pursuit trials). It should be noted that, our results of similar self-motion performance between AD patients and healthy controls during passive viewing are seemingly contradictory with previous studies showing that AD is associated with elevated optic flow threshold^5, 36^. However, this difference may well be explained by the methodological differences between studies. More precisely, the previous studies varied the coherence levels of optic flow (by adding randomly-moving dots) and took the coherence threshold as a measure of perceptual capability. In contrast, in the current study we did not incorporate visual noise into the optic flow. Instead, we varied the angle of optic flow to obtain the psychometrical performance. To what extent the perceptual performance based on these two different measures are related is still unknown.

There is a possibility that between-group difference of optic flow perception in pursuit conditions was a consequence of differences in the qualities of pursuit movements per se. This explanation is also compatible with the apparent lack of between-group difference in fixation trials. Since we did not conduct simultaneous eye-tracking to monitor gaze positions in parallel to the psychophysical measurements, we cannot completely exclude this possibility. More-carefully designed experiments are needed to better determine the nature of these compensation differences. Another limitation of the current study is that, due to a lack of sufficient variability in MMSE scores, we could not know whether the amounts of pursuit compensation impairment are correlated with the severity of AD pathologies. Future studies should recruit more AD patients with larger MMSE variability (from early to late stages) to explore the correlative relationships between compensation deficits and AD pathology.

The current study has implications on the cortical dysfunctions in AD pathology. Optic flow is generally believed to be processed by the dorsal subdivision of medial superior temporal area (MSTd) and ventral intraparietal area (VIP)^37^. Neurons in these two areas received not only visual information about the optic flow^38–40^ but also extra-retinal information including signals related to pursuit eye movements^41–43^. Single-unit neurophysiologic studies in monkeys have shown that neural responses in MSTd and VIP are stable despite of distorted retinal optic flow in the presence of eye movements^44–46^, indicating the involvement of these two areas in movement compensation. Our results of impaired pursuit compensation during optic flow perception in AD patients suggest that AD pathology is probably associated with the progressive cell death and/or synaptic loss in these two parietal areas. However, this does not exclude the possibility of structural and functional alterations of cerebral white matter in AD, as the integrity of white matter is also pivotal for normal brain functions^47, 48^. Further studies with animal models of AD are needed to advance our understanding of its underlying cortical and white matter pathology and to explore the relevance of eye movement compensation deficits in AD.
